# Psychoeducational interventions in adolescent depression: A systematic review

**DOI:** 10.1016/j.pec.2017.10.015

**Published:** 2018-05

**Authors:** Rhys Bevan Jones, Anita Thapar, Zoe Stone, Ajay Thapar, Ian Jones, Daniel Smith, Sharon Simpson

**Affiliations:** a Child & Adolescent Psychiatry Section, Division of Psychological Medicine & Clinical Neurosciences, Cardiff University, Wales, UK; b Division of Psychological Medicine & Clinical Neurosciences, Cardiff University, Wales, UK; c Institute of Health and Wellbeing, University of Glasgow, Scotland, UK; d Tâf Riverside Practice, Cardiff, Wales, UK

**Keywords:** PI, psychoeducational intervention, Psychoeducation/education, Adolescence, Depression, Prevention, Treatment

## Abstract

**Background:**

Adolescent depression is common and leads to distress and impairment for individuals/families. Treatment/prevention guidelines stress the need for good information and evidence-based psychosocial interventions. There has been growing interest in psychoeducational interventions (PIs), which broadly deliver accurate information about health issues and self-management.

**Objective, methods:**

Systematic search of targeted PIs as part of prevention/management approaches for adolescent depression. Searches were undertaken independently in PubMed, PsycINFO, EMBASE, guidelines, reviews (including Cochrane), and reference lists. Key authors were contacted. No restrictions regarding publishing dates.

**Results:**

Fifteen studies were included: seven targeted adolescents with depression/depressive symptoms, eight targeted adolescents ‘at risk' e.g. with a family history of depression. Most involved family/group programmes; others included individual, school-based and online approaches. PIs may affect understanding of depression, identification of symptoms, communication, engagement, and mental health outcomes.

**Conclusion, practice implications:**

PIs can have a role in preventing/managing adolescent depression, as a first-line or adjunctive approach. The limited number of studies, heterogeneity in formats and evaluation, and inconsistent approach to defining PI, make it difficult to compare programmes and measure overall effectiveness. Further work needs to establish an agreed definition of PI, develop/evaluate PIs in line with frameworks for complex interventions, and analyse their active components.

## Highlights

• Guidelines stress need for good information and psychosocial interventions.
• Depression PIs include family/group, individual, school and online programmes.
• PIs may affect: understanding, identification, family communication, mental health.
• Limitations: only a few PIs evaluated, heterogeneity, inconsistent definition of PI.
• Future work: develop/evaluate PIs in line with frameworks for complex interventions.

## 1 Introduction

Depression is common in adolescence, and leads to distress for the young person and their family/carer. It is associated with social and educational impairments. It also predicts suicide, deliberate self-harm and poor physical health, and can mark the beginning of long-term mental health difficulties [1]. Early treatment and prevention of adolescent depression is therefore a major public health concern [2]. However, depression is difficult to recognise and treat in this age group, and engaging young people in prevention and early intervention programmes is a challenge for health and other services [3].

Guidelines for depression in young people (e.g. National Institute for Health and Care Excellence (NICE) [4]; American Academy of Child and Adolescent Psychiatry (AACAP) [5]) stress the need for good information and evidence-based psychosocial interventions for the young person, family and carer. Psychosocial interventions are likely to be important in young people for promoting resilience and preventing relapse [1], [6]. Whilst the risk factors and possible causes of adolescent depression are complex, individuals with a family history of depression and psychosocial stress are known to be at a higher risk, and could be targeted for such strategies, along with those with a history of depression [1].

Over recent years there has been growing interest in psychoeducational interventions (PIs); that is the delivery of accurate information to individuals, families and carers about mental health or a specific diagnosis (including possible causes and symptoms), management (including associated risks/side-effects) and prognosis, and how affected individuals can stay well [5], [7], [8], [9]. Much of the literature on PIs has been in relation to individuals with schizophrenia and bipolar disorder and their families/carers [5], [7], [10], although there has been increasing interest in depression. Findings from a recent systematic review concluded that PIs are effective in improving the clinical course, treatment adherence, and psychosocial functioning of adults with depression [11].

However, there is no published review on PIs in the prevention and management of adolescent depression. This is an important knowledge gap; depression is more common than bipolar disorder and schizophrenia, and the presentation and management of depression is different in young people compared to adults, as might be their response to PIs. Further investigation could have implications on clinical practice, by informing the way in which practitioners communicate with young people and families/carers regarding depression (and future resources, interventions and guidelines), and raising public awareness of adolescent depression.

A systematic review was conducted of the published literature on PIs for adolescents with (or at high risk of) depression. The aim of the review was to i) systematically search and review the literature investigating PIs in the context of adolescent depression; ii) describe the range of PI programmes; iii) summarise the evidence for the effectiveness of different programmes.

## 2 Methods

### 2.1 Selection criteria

Inclusion criteria were: studies of PIs (as defined earlier) targeting depression as part of prevention or management approaches in the adolescent age group (studies were included if at least some of the participants were between 12 and 18 years old); targeted programmes for individuals with depression/depressive symptoms (which could include relapse prevention) OR those at high-risk, and/or their families/carers. Studies were included only if there was evaluation of the response of adolescents or families/carers (no other groups, e.g. teachers), with quantitative or qualitative methodology.

Articles were restricted to those published or translated into English. Articles were also considered if only elements of the published study were of relevance (e.g. if the control group in a trial was given a PI).

Exclusion criteria were as follows: only adults or young children, other mental disorders only (including bipolar disorder), non-psychiatric disorders, established therapeutic approaches alone (including cognitive behavioural therapy (CBT)) or no evaluation of the programme. Universal programmes or general health information/education (e.g. in printed leaflets) were not considered. Single case reports/studies were excluded, but otherwise there were no restrictions on the format of the PI, study design, presence of a comparison/control group, or length of follow-up. This inclusive approach to the search was taken, as the initial search for PI randomised controlled trials (RCTs) returned a small number of papers.

### 2.2 Search strategy

**Fig. 1:**
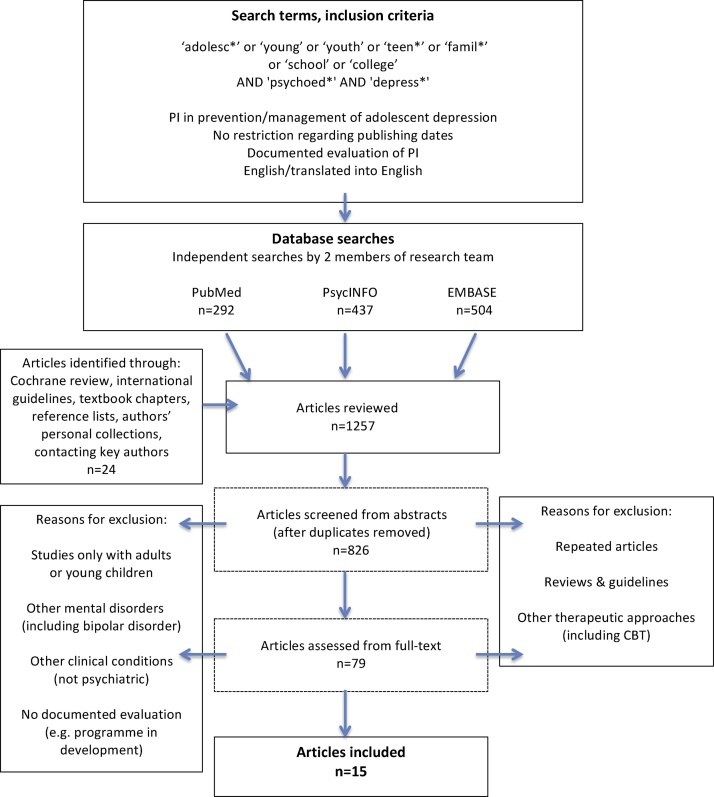
Flow diagram: Methodology for article selection.

Searches were conducted in PubMed, PsycINFO and EMBASE by two independent investigators (RBJ, ZS). Search terms included ‘adolesc*’ or ‘young’ or ‘youth’ or ‘teen*’ or ‘famil*’ or ‘school’ or ‘college’ AND ‘psychoed*’ AND ‘depress*’ in the title or abstract, with no restriction regarding publication dates (Fig. 1, flow diagram). These searches were performed up to January 2017.

Articles were also identified through reference lists and the authors’ personal collections, including studies in a Cochrane review [12]_,_ international guidelines [4], [5], chapters in relevant textbooks [13], and educational material (e.g. Royal College of Psychiatrists (RCPsych), NICE, AACAP, Black Dog Institute, Orygen Youth Health). Key authors with expertise in adolescent depression interventions were contacted, and authors were contacted for details on studies (e.g. regarding participants and follow-up).

### 2.3 Study selection, data extraction

Data extracted from the studies was tabulated independently by the authors, with columns including mode of delivery of PIs, study design, participant characteristics, assessment/follow-up, and findings. RBJ and ZS independently reviewed all the abstracts and extracted data, and discussed the studies to be included in meetings following the independent searches. Where there was disagreement, both researchers reviewed the studies together before deciding on whether they were included. Other authors (SS, AT, DS, AKT, IJ) were consulted where there was uncertainty or disagreement.

The review was guided by the PRISMA statement [14], and the risk of bias by Cochrane Collaboration guidelines [15].

## 3 Results

### 3.1 Description of PIs

The findings from the review suggested there are many ways in which PIs for adolescent depression can be delivered and categorised, broadly ranging from ‘one-to-one’ approaches to multi-group and family approaches. Programmes can vary according to the number of participants (individual, family, group), setting (community, school, service), and mode of communication (printed, online, game, lecture). Several formats and approaches can also be adopted by a single programme.

PIs have been described as passive or active [16]. However, the literature search suggested that programmes may be a mixture of both, for example online interventions could communicate information ‘passively’, but with interactive components and access to therapists or forums. A similar approach was used to categorise studies according to whether programmes were ‘proximal’/‘in person’ or ‘long distance’ (e.g. online, mail-outs). They could also be described according to whose response was evaluated – young person, family/carer, other (e.g. teacher), or a combination.

Fifteen studies were identified that met the inclusion criteria. In the following sections, the studies are presented according to whether they targeted adolescents with a current diagnosis of depression or depressive symptoms, and/or their families (some included relapse prevention) (seven studies, Table 1), or whether they focused on adolescents ‘at risk’ (eight studies, Table 2), for example if there was a family history of depression. PIs were also subcategorised according to whether they took a predominantly family/group (10 studies), individual (two studies), or online approach (three studies). All ‘in person’ studies in the review (12 studies) involved a professional as a facilitator, whilst the ‘long distance’ studies (three studies) did not.

**Table 1:** Studies of psychoeducational interventions (PIs) for adolescents with depression, and families/carers.

Study, country	Details of Intervention (and control)	Study Design	Participant characteristics (n)	Assessment, follow-up	Findings
Family PI					
Sanford et al. [17], Canada	**Intervention:**	Randomised controlled trial	31 adolescents aged 13–18 years (20 females: 11 males), meeting DSM-IV criteria for MDD, and their families.	Primary outcomes:	Intervention improved RADS, SSAI, ACL post-treatment and follow-up, compared to usual treatment: effect size >0.5 for all.
	Adjunctive PI: group sessions with all family members at home, PLUS usual treatment.	(feasibility, effectiveness)	(16:15)	Reynolds Adolescent Depression Scale (RADS); Structured Social Adjustment Interview (SSAI) (adolescent social functioning); Family Assessment Device (FAD) (family functioning); Adjective Checklist (ACL) (adolescent-parent relationship)	Effect size for RADS on follow-up: 0.64.
	Twelve structured interactive 90-min sessions, with manual. Aims: increase family knowledge about depression, appreciate effects on family, improve communication between adolescent and family and coping strategies.			Secondary outcomes:	Greater satisfaction reported with intervention.
	****Control:****			Children’s Global Assessment Scale (CGAS) (adolescent); Client Satisfaction Questionnaire (CSQ) (parent satisfaction with services)	
	Usual treatment: individual/group counselling.			Baseline (plus retest at 2 weeks), 3 months (mid-treatment), 6 months (post-treatment), 9 months (follow-up)	
Lopez et al. [18], USA	**‘Children’s Medication Algorithm Project (CMAP)’:**	Feasibility trial with 2 arms	90 children aged 6–17 years (26 females: 64 males) with diagnosis of depressive disorder, ADHD or both, being treated with medication in 4 community clinics.	Parent Satisfaction Questionnaire; Child/Adolescent Satisfaction Questionnaire; CMAP Education Log	Majority of caregivers (63%) and children (60%) happy with amount of information and found this helpful. 20% of parents and 14% of children/adolescents received much more information than they wanted. 90–100% of children and parents found written materials helpful.
	Group intervention facilitated by clinicians/assistants, with manual, on medication, self-monitoring, lifestyle, coping strategies.			Baseline, then 4-month intervals	Programme successfully implemented, but follow-up data not analysed (confirmed from personal communication with authors).
	Programme structured but could be tailored to families’ needs. Several available formats. No fixed number of sessions (median:6).				
	Aims: improve compliance with medication and coping strategies.				
Brent et al. [19], USA	2-h session with manual, for parents on diagnosis, course, treatment, methods of coping with family member. Depression described as a chronic, recurrent illness.	Trial of acceptability, feasibility, efficacy	62 parents of 34 adolescents (22 females: 12 males) with mood disorder (primarily depressive disorder).	Questionnaire on attitude and knowledge about depression, and views of the programme Baseline, post-intervention	Improvement in knowledge, modification of dysfunctional beliefs about depression and treatment.
					‘Significant improvement’ on 8/21 questionnaire items, decline in one item.
					Useful, interesting for almost all (97%) participants.

INDIVIDUAL PI					
Parker et al. [20], Australia	**Simple low-intensity interventions.**	Factorial (2 × 2) randomised controlled trial	176 help-seeking 15–25 year olds (mean age 17.6 years) with sub-threshold or mild-moderate depression/anxiety.	Primary outcomes:	Reduction in depression symptoms in BAPA and PI groups, greater reduction with BAPA, but not anxiety symptoms. Effect size post-intervention: BDI-II: d = 0.41 (95% CI 0.07–0.76); MADRS: d = 0.48 (95% CI 0.13–0.82).
	Up to 6 manualised weekly sessions.	(acceptability, effectiveness)	Lifestyle PI:86 (53 females:33 males), BAPA:88 (53 females:35 males)	Beck Depression Inventory-II (BDI-II); Montgomery-Asberg Depression Rating Scales (MADRS); Beck Anxiety Inventory (BAI)	Lifestyle PI:
	Exercise: Behavioural activation physical activity (BAPA) v **Lifestyle psychoeducation** (e.g. physical activity, sleep, substance use)			Secondary outcomes:	BDI-II (mean): Baseline: 22.23, Post-intervention 14.09;
	Psychological: Problem Solving Therapy v Supportive Counselling.			Clinical caseness; Substance (use) and Choices Scale; Social and Occupational Functional Scale; Active Australia (physical activity) Survey; Questionnaire on other interventions received	MADRS (mean): Baseline: 20.44, Post-intervention 12.87;
				Baseline, post-intervention	BAI (mean): Baseline: 15.56, Post-intervention 7.88.
					Problem solving therapy not superior to supportive counselling. No interactions between interventions.

COMPUTERISED/ONLINE PI					
Stasiak *et al.*[21], New Zealand	**Intervention:**	Randomised controlled trial	34 adolescents (13–18 years) with low mood (14 females: 20 males), self-referred to school counsellors across 8 urban schools.	Primary outcome: Child Depression Rating Scale Revised (CDRS-R);	Reductions in depression scores in both groups, greater reduction with cCBT.
	**‘The Journey’**, computerised CBT programme (cCBT) at school, with guidebook. No school counsellor support unless requested.	(feasibility, acceptability, effectiveness).	(17:17)	Secondary outcomes: RADS-2; Pediatric Quality of Life Inventory (PedsQL); Adolescent Coping Scale (ACS)	CDRS-R mean change:
	7 × 25–30 min multimedia modules (‘fantasy game-like environment’), on problem solving, conflict resolution, identifying and challenging unhelpful thoughts, relaxation techniques.			Acceptability: Brief satisfaction questionnaire	cCBT = 17.6 (CI = 14.13–21.00); CPE = 6.1 (CI = 2.01-10.02); p < 0.001. Effect size between groups: 1.7.
	**Control:**			Baseline, post-intervention, 1-month follow-up	CPE had been helpful, positive feedback on computer-based format. Some felt it was more suited to younger ages.
	**Psychoeducation computer programme (CPE)**. Same structure as above but different content − on depression, ‘mental health hygiene’, stress reduction. CPE more ‘instructional’ than ‘therapeutic’.				
Demaso et al. [22], USA	**‘Depression Experience Journal (EJ)’**: website for children/adolescents, families and healthcare professionals to share personal experiences of living/working with mental illness (to ‘facilitate healthy coping’). Used individually or with others.	Development trial − feasibility, safety	38 primary caregivers, each with a child aged 8–19 years (26 females: 12 males) with depression, during a psychiatric hospital admission.	2 semi-structured interviews:	Parents satisfied overall with EJ and presentation of stories and facts. Personal stories most helpful.
				First assessed families’ experiences of child’s depression and management;	They suggested greater number and wider variety of narratives, and more interactivity.
				Second on views of intervention: using satisfaction & safety, concerns/areas for improvement, specific impacts, coping response & attitude change scales.	
				Baseline, 2–4 weeks after use	
Stjernswärd & Hansson [23], Sweden	Web-based support for relatives of individuals with depression − psychoeducation module, diary, forum.	Explorative open trial	25 relatives of individuals (including adolescents) with depression.	System usability scale (questionnaire); Content analysis of forum	Generally well-received.
					Intervention could help e.g. with feelings of isolation.

**Table 2:** Studies of psychoeducational interventions (PIs) in adolescents at high-risk of depression, and families/carers.

Study, country	Details of Intervention (and control)	Study Design	Participant characteristics (n)	Assessment, follow-up	Findings
Family PI (parental depression)					
Beardslee et al. [26], USA	**Intervention:**	Randomised controlled trial	37 families, each with an asymptomatic (non-depressed) 8–15 year old child (53 children in total, 21 females: 32 males), and at least one parent who had experienced a mood disorder (primarily depression) within 18 months	Semi-structured Interview about Disorder Impact and Intervention (parent) (SII) (family functioning; illness-related behaviour; benefits from intervention)	Intervention parents:
	Preventive group intervention facilitated by clinicians, with manual. 6–10 sessions (mean 7.7) attended mainly by parents; adolescents attended at least one clinician meeting and one family meeting.	(‘First-phase pilot study’)	(19:18)	Semi-structured Child Interview (SCI) (functioning; knowledge, feelings, experience of parent depression; coping style, perception of change)	- happier with factual information received than controls. - reported greater understanding of their feelings about mental illness and increased marital support.
	Main concepts: increased familial understanding of the disorder, appreciation of children’s experience of parental illness and potential effects.			Baseline, post-intervention (8.6 weeks on average)	Improved communication with children about their illness because of increased understanding in parent and child.
	**Control:**				
	2 × 1-h lectures to small groups, attended by parents only − on depression, its effects and warning signs.				
Beardslee et al. [27], USA	See details above	Randomised controlled trial (Efficacy study to establish sustained effects)	See details above	Semi-structure interviews as above Second follow-up, 1.5 years after enrolment	Intervention parents reported more positive changes than controls. Scores similar to those recorded post-intervention, which demonstrated sustained effects.
Beardslee et al. [28], USA	As above		First 12 families to complete intervention above	As above; clinical case discussions	Healing elements identified included:
	Authors explored ‘healing principles’ that contributed to positive changes in family behaviour and attitudes, which in turn enhanced resilience in children.			Follow-up (at least 3 years)	- demystification of illness, - modulation of shame and guilt, - increase in capacity for perspective taking, - development of hopeful perspective and belief in own competence.
					Families developed shared understanding of illness.
Beardslee et al. [29], USA	As above	Randomised controlled trial	93 families (121 children, 52 females: 69 males), same criteria as above	Schedule for Affective Disorders and Schizophrenia-Lifetime Version (SADS-L) & Streamlined Longitudinal Interval Continuation Evaluation (SLICE).	PI had long-term effects on how families address problems regarding parental mental illness. Parents found intervention more beneficial than lecture in changing child-related behaviour and attitudes.
	Telephone contacts/refresher meetings at 6–9 month intervals, with psychologists, social workers, nurses.	(‘Large-scale efficacy trial’)	(53:40)	Schedule for Affective Disorders and Schizophrenia for School-Age Children, Epidomologic Version Revised (Kiddie-SADS-E-R) & Kiddie-Streamlined Longitudinal Interval Continuation Evaluation (K-SLICE).	Children reported increased understanding of parental illness over lecture group (x^2^_1_ = 8.2, p = 0.004).
	Educational material linked to family’s experience, reducing feelings of guilt/blame and helping children to build relationships within/outside of home.			Global Assessment Scale (GAS).	All children reported reduced depressive symptomatology over 2 years since intervention. (x^2^_1_ = 7.3, p = 0.007), but no significant effect of group on this change (x^2^_1_ = 0.2, p = 0.69).
				Youth Self-Report (YSR).	
				SII & SCI (see previous Beardslee et al. studies)	
				Baseline, post-intervention, 1 and 2 years after enrolment	
Solantaus et al. [30], Finland	**Intervention:**	Randomised controlled trial	109 parents with a mood disorder (primarily depression) and their partners, who had at least one child aged 8–16 years (not treated for psychiatric disorder)	Beck Depression Inventory (BDI); Spielberger State Anxiety Inventory (STAI); Strengths and Difficulties Questionnaire (SDQ); Screen for Child Anxiety Related Emotional Disorders (SCARED).	In both groups:
	**‘Family Talk Intervention (FTI)’** preventive programme, included psychoeducation. Minimum 6 sessions (more for families with >1 child), with manual. 2 parent-focussed sessions followed by session with each child − on depression, how to talk about it with family members, coping with family problems and answering children’s questions.	(‘Efficacy study’)	(53:56)	Baseline, 4, 10 and 18 months post-intervention.	- improvement seen in children’s prosocial behaviour - reduction in emotional symptoms and anxiety.
	**Control:**				Changes noted sooner with FTI (baseline-4 months) than LT (4–10 months). No group differences after 10–18 months follow-up.
	**Let’s Talk about the Children (LT)’**: brief, child-focussed, discussion with parents to assess child’s situation and support them. Duration: single 15-min session to 2 × 45-min sessions.				Marginal decrease in hyperactivity in both groups.

**Family PI (psychososocial stress)**					
Jordans et al. [31], Burundi	Intervention:	Controlled trial	120 children aged 10–14 years with high levels of psychosocial stress on screening due to political violence (and their parents)	Primary outcomes: Depression self-rating scale (DSRS); Aggression Questionnaire.	Intervention parents saw improvement in child’s aggression, effect size d = 0.60 (p < 0.001), especially in boys.
	Group-based parenting programme, adapted from manual for parents about helping children cope with political violence.				
	Facilitated by 2 community counsellors (attended by parents only). 2 sessions: First (2.5 h) on problems affecting children and communication, second (3 h) on how to manage difficulties.		(58 (32 females: 27 males): 62 (39 females: 23 males))	Secondary outcome: Family Social Support scale	No improvement in depressive symptoms or perceived family support.
	Part of larger mental health package for low and middle-income countries.			Baseline, 3-weeks post-intervention	Majority of parents satisfied with intervention, and learned to be ‘better parents’.
	**Control:** Waiting list.				
Martinez-Pampliega et al. [32], Spain	**Intervention:**	Controlled trial	34 parents, total of 51 children (31 females: 25 males), aged 2–23 years (including 6 family controls).	Child Behavior Checklist (CBCL); Symptoms Checklist (parental) (SCL-90); O’Leary-Porter Scale of Marital Conflict (OPS); Family Communication Scale.	Differences, especially in follow-up, in perceived family conflict (d = 0.85, p = 0.01) and children’s mental health symptoms: anxiety/depression (d = 0.57, p < 0.001) and aggression (d = 0.65, p < 0.001).
	**‘Egokitzen’**: Post-divorce parent intervention. 11 weekly (1.5 h) sessions, with role-playing, debates, group activities - on divorce, interparental conflict, parenting styles and discipline.	(exploratory, ‘quasi-experimental’)		Baseline, post intervention, 6-months follow-up	
	**Control:** Waiting-list				

INDIVIDUAL PI					
Barnet et al. [33], USA	**Intervention**:	Randomised controlled trial	84 pregnant adolescents aged 12–18 years (predominantly with low incomes and African-American), from urban prenatal care sites.	Adult-Adolescent Parenting Inventory (AAPI); Center for Epidemiologic Studies Depression (CES-D); School status − self-report.	Intervention improved parenting attitudes (by 5.5 points higher than controls (95% CI 0.5–10.4, p = 0.3)) and school continuation (3.5 times greater than control, 95% CI 1.1-11.8, p < 0.05).
	Community-based programme for adolescent mothers. Trained home visitors paired with mothers through child’s second birthday.		(44: 40)	Baseline, 1 and 2 years follow-up	Did not reduce odds of repeat pregnancy or depression, or achieve coordination with primary care.
	Parenting curriculum − encouraged contraceptive use, connected adolescent with primary care, promoted school continuation.				
	Rationale: Adolescent mothers at risk for rapidly becoming pregnant again, depression, school dropout, and poor parenting.				
	**Control**: Usual care.				

Within each subcategory, the studies are presented according to a hierarchy of evidence, with RCTs presented first and small-uncontrolled studies discussed last. Eight of the studies were RCTs, and the risk of bias is presented in Table 3 [15]. The outcomes of interest included understanding, attitude, behaviour change, (family) communication and support, and mental health outcomes (depression, anxiety, aggression).

**Table 3:** Presentation of risk of bias for randomised controlled trials (RCTs) in the review.

	Random sequence generation (selection bias)	Allocation concealment (selection bias)	Blinding of participants & personnel (performance bias & detection bias)	Blinding of outcome assessment (performance bias & detection bias)	Incomplete outcome data (attrition bias)	Selective reporting (reporting bias)
Sanford et al. [17]	+	+	−	−	+	?
Parker et al. [20]	+	+	−	+	+	+
Stasiak et al. [21]	+	+	+	?	+	+
Beardslee et al. [26]	+	?	−	?	+	?
Beardslee et al. [27]	+	?	−	?	+	?
Beardslee et al. [29]	+	?	−	?	+	?
Solantaus et al. [30]	+	?	−	?	+	?
Barnet et al. [33]	?	?	−	?	+	?

Key: +: low risk of bias; −: high risk of bias; ?: unclear risk of bias.

### 3.2 Adolescents with depression: PIs for adolescents and families/carers (seven studies, Table 1)

#### 3.2.1 Family PI

Sanford et al. [17] carried out a pilot RCT of a programme, comparing the effectiveness of twelve structured sessions conducted at home, with usual treatment for adolescents meeting DSM-IV criteria for major depressive disorder. Sessions aimed to increase family knowledge about depression, understand effects and improve communication and coping strategies. Sixteen adolescents (aged 13–18 years) and their families participated in the intervention group, and 15 in the control (individual or group counselling). Assessments were done at baseline and three-monthly intervals up to nine months. The programme improved adolescent social functioning, family relationships, depressive symptoms (effect size >0.5 for all, 0.64 for depressive symptoms (RADS scale) on follow-up) and duration of remission, and participants reported greater satisfaction compared to counselling.

The ‘Children’s Medication Algorithm Project’ targeted young people with depressive disorder, ADHD or both (diagnosed by the treating psychiatrist) – to improve compliance with medication (and self-monitoring) and coping strategies [18]. The information was general at first, but then tailored to families’ needs and developmental age, and available in different formats. There was no fixed number of sessions (median = 6). Ninety participants (aged 6–17 years) were recruited from community clinics, and asked to complete surveys at baseline and after four months. The majority of caregivers (63%) and children (60%) were happy with the amount of information and found it helpful. The authors reported (in personal communication) that the programme was completed, but follow-up results were not analysed.

Brent et al. [19] described a feasibility study of a programme for parents of adolescents with depression, cited in AACAP parameters. This consisted of a session on diagnosis (depression as a chronic, recurrent illness), course and treatment, and methods of coping with a family member with depression. Sixty-two parents and 34 adolescents participated. There was an improvement in their knowledge, and modification of dysfunctional beliefs about depression and treatment. Almost all participants (97%) described this as useful and interesting.

#### 3.2.2 Individual PI

PI was provided to a control group in a factorial RCT to evaluate low-intensity interventions in young people, with mild-moderate depression and/or anxiety [20]. The mean age of participants was 17.6 years (range 15–25 years). The group was delivered ‘lifestyle’ PI, particularly on physical activity, sleep and substance use, by psychologists in six manualised weekly sessions. Eighty-six young people participated in this group, whilst 88 received the ‘intervention’, behavioural activation physical activity (BAPA). Depressive symptoms reduced in both groups, but BAPA was more effective (effect size: BDI-II d = 0.41, MADRS d = 0.48), although there was no reported follow-up.

#### 3.2.3 Computerised/Online PI

A school-based RCT included a ‘psychoeducation computer programme’ (CPE) in the control arm, versus the main intervention, a computer ‘fantasy game’ with CBT content, ‘The Journey’ [21]. The latter comprised seven modules, including ones on problem solving, conflict resolution, challenging unhelpful thoughts and relaxation techniques. CPE had a similar structure, but was more **‘**instructional’ than therapeutic, and covered depression, ‘mental health hygiene’ and stress reduction. Seventeen adolescents (aged 13–18 years) with low mood participated in each group, referred from school counsellors. These were assessed at baseline, post-intervention and one month. Reductions in depression scores (CDRS-R scale) were seen in both groups, but greater in the CBT group (effect size between groups: 1.7). Participants reported the CPE had been helpful and favourable feedback on the computer-based format.

The ‘Depression Experience Journal' website served as a platform for children/adolescents, families and professionals to share their personal experiences of living/working with mental illness [22]. This aimed to “facilitate healthy coping”, and could be used individually or with others. A feasibility trial included 38 primary caregivers, each with a child aged 8–19 who had been admitted to hospital because of mental health difficulties. Assessments were done at baseline and 2–4 weeks after use. Parents were satisfied overall with the way information was presented, and personal stories were most helpful. They suggested increasing the number/range of narratives, and making the site more interactive.

Stjernswärd and Hansson [23] conducted an exploratory trial of a web-based supportive intervention for relatives of those with depression. This included a psychoeducation module, diary and forum. One of the discussion themes was the youth to adult transitions. The 25 participants included parents and other relatives of young people with depression, or those who had suffered from a young age, although the authors noted (in personal communication) it was unclear how many were adolescents. The tool was generally well-received, and highlighted how web-based support could help with feelings of social isolation.

### 3.3 Adolescents at risk of depression: PIs for adolescents and families/carers (eight studies, Table 2)

A family history of depression is one of the best-known risk factors for adolescent depression [24]. Children of depressed parents are therefore a potential target group for depression prevention programmes. Another major risk factor for depression in young people is psychosocial stress, which is another consideration when developing prevention programmes [25].

#### 3.3.1 Family PI where there is parental depression

Five studies focused on adolescents at risk because they had a parent with depression. Beardslee et al. [26] described an intervention targeting parents with depression and their asymptomatic child. It consisted of 6–10 sessions facilitated by clinicians, and the main concepts were increased familial understanding of the disorder, and appreciation of children’s experience of parental illness. The pilot study included 19 families in the intervention group, 18 in the control (lectures on depression, its effects and warning signs), each with an 8–15 year-old child. Assessments were done at baseline and after eight weeks. Adults in the intervention group were happier with the information received, and reported greater understanding of their feelings about mood disorders and increased marital support. There was also improved communication with their children about their illness because of increased understanding in both parent and child.

The authors established sustained positive effects on these outcomes 1.5 years after enrolment [27], and identified specific ‘healing principles’ that contributed to the changes in family behaviour and attitudes, which enhanced resilience in children [28]. These principles were based on findings from the first 12 families to complete the intervention, and included demystification of the illness, modulation of shame/guilt, increase in the capacity for perspective taking, development of a hopeful perspective and a belief in one's own competence.

AACAP parameters referred to a RCT by Beardslee et al. [29] with the same recruitment criteria. The study design was updated with telephone contacts/refresher meetings carried out at 6–9 month intervals. There was focus on linking the educational material to a family’s individual experience, reducing feelings of guilt/blame, and helping children learn to build relationships within and outside the home. Fifty-three families participated in the intervention arm, and 40 in the ‘lecture’ control group. Assessments were made at baseline, post-intervention, after 1 and 2 years. There were long-term effects on how families address problems regarding parental mental illness. Parents reported that the intervention was more beneficial than the lecture in changing child-related behaviour and attitudes. Children reported increased understanding of parental illness and reduced depressive symptomatology (x^2^_1_ = 7.3) after the intervention over two years.

PI was a substantial component of the ‘Family Talk Intervention’ (FTI) [30]. This consisted of a minimum of six sessions, and guidebooks were provided to participants. Two parent sessions were followed by one with each child. Parents were taught about depression, and how to talk about it with family members, cope with family problems and answer children’s questions. In an RCT, 53 parents treated for a mood disorder (and partners) participated in FTI, and 56 (controls) underwent ‘Let’s Talk about the Children (LT)’, a brief PI parent discussion to assess/support the child (aged 8–16). They completed questionnaires at baseline and 4, 10 and 18 months post-intervention. An improvement was seen in children’s prosocial behaviour and reduction in their emotional symptoms and anxiety in both groups, although they were noted earlier in the group who received FTI.

#### 3.3.2 Other family PI

Two family studies targeted adolescents at elevated risk of depression due to psychosocial stress exposure. Jordans et al. [31] conducted a controlled (pilot) trial of a parenting group PI. This was part of a larger mental health package for low and middle-income countries (LMIC), and targeted families reporting high levels of psychosocial stress due to political violence. Sessions focused on communication, problems affecting children and how to manage them. Fifty-eight children aged 10–14 years (and their parents) were recruited from the ‘treatment school’, and 62 from the ‘control school’ (waiting list for intervention). Assessments were made at baseline and three weeks post-intervention. No improvement was seen in child depressive symptoms or perceived family support, although parents in the intervention arm saw an improvement in their child’s aggression (effect size d = 0.6), especially in boys. The majority of parents reported they were satisfied and had learned to be ‘better parents’.

A controlled (exploratory) trial of ‘Egokitzen’, a post-divorce intervention for parents and their children, comprised 11 weekly sessions on divorce, interparental conflict, and parenting styles/discipline [32]. Thirty-four parents and 51 children (aged 2–23 years) participated, and six parents and nine children were in a comparison group (waiting list). They did not state how many adolescents participated, although eight were over 13 years old. There was some effect of the intervention on the children’s mental health symptoms (anxiety/depression: d = 0.57, aggression: d = 0.65), particularly in the 6-month follow-up.

#### 3.3.3 Individual PI

Barnet et al. [33] described a RCT of a community-based home-visiting programme for adolescent mothers. The authors noted how this group was at risk of becoming pregnant again, depression, school dropout, and poor parenting. Home visitors were paired with each adolescent through the child’s second birthday, and delivered a parenting curriculum, encouraged contraceptive use, connected the adolescent with primary care, and promoted school continuation. Forty-four adolescents (aged 12–18 years) were in the home-visited group, and 40 in a control group (usual care), predominantly with low incomes and of African-American origin. Structured interviews were done at baseline and 1 and 2 years’ follow-up. This programme improved adolescent mothers’ parenting attitudes and school continuation, but it did not reduce their odds of depression or repeat pregnancy, or achieve coordination with primary care.

## 4 Discussion and conclusion

### 4.1 Main findings

This is the first systematic review of PIs in the prevention and management of adolescent depression. The main objective was to identify studies on PIs for adolescents with, or at high risk of, depression, by rigorous methods, to explore the content and design of existing programmes and to evaluate their effectiveness. This could help inform clinical practice and the development of future programmes and guidelines, and increase awareness of adolescent depression.

Fifteen PI studies for adolescent depression were identified in this review. The studies showed a range of approaches to PI, and the vast majority were ‘in person’ (‘proximal’) and ‘active’, and most involved content presented to families/groups facilitated by a professional. NICE [4] and AACAP parameters [5] state that the involvement of the family is important in the management of adolescent depression, the motivation for treatment often comes from parents, and any parental and child mental health difficulties should be treated in parallel.

Whilst only a few studies in the review were categorised as ‘individual’ or ‘group’ PI, many of the studies in other categories, such as ‘family’ or ‘computerised’ PI, embraced one-to-one or group approaches. This demonstrated how programmes could incorporate a range of formats to engage/communicate information, consistent with ‘blended learning’ approaches [34].

There is emerging literature on computerised and online interventions, although many were not included in the review because there was no evidence they had been evaluated [35], [36].

Some of the studies in the review [21], [31], recruited adolescents via schools. Most school-based programmes found, however, were universal mental health programmes [37], [38], and were not included in the review. There were also programmes developed for teachers only [39], and assessments of mental health literacy [40], but not in association with PI.

PIs for mental health difficulties other than depression were not included in the final review, but could help inform future programmes. For example, elements of PIs for bipolar disorder [10], [41], anxiety and suicide [42], could be examined, particularly where dealing with depressive symptoms. There were also case studies of depression programmes [e.g. 43], which could be explored with more participants.

### 4.2 Effectiveness of PI

In general terms, PIs aim to inform and empower users to make decisions about their welfare and care, and promote resilience. In the current review, studies showed PIs may have a beneficial effect on a range of measures, including knowledge/understanding of depression and its effects, behaviour and attitudes, treatment adherence, and depression and other mental health and wellbeing outcomes. Increased parental and child understanding which may be facilitated by PIs, can lead to improved communication, conflict resolution and problem-solving, and this appears to be important in managing/preventing depressive symptoms in adolescence [29].

Evidence for the effectiveness of PIs is limited, but based on the evidence to date PIs in adolescent depression show some promise, although further well-designed multi-centre trials are needed. All this is consistent with a review of PIs for depression in adults, which concluded that whilst few studies have been published in this field, PIs can help improve the clinical course, treatment adherence and psychosocial functioning in adults, and family PI is seen as part of its ‘optimal treatment’ [11].

As with all interventions, it is important to consider possible side-effects of PIs. Adolescents with depression can experience difficulties with concentration, energy levels and motivation [44]. Some studies included in the review noted that research participants stated there was too much information in the programmes [e.g. 18]. Detailed health information could make the individual, family and carers anxious and distressed, or lead to excessive ‘self-checking’ and rumination. There may also be a risk of dependence on, or over-compliance with, the PI facilitator, or at the other extreme, an over-reliance on self-management strategies.

At this time it is not possible to conclude that PIs are effective in adolescent depression given the small number of studies and the variable methodological quality. For example, there was potentially a high risk of performance and detection bias in many of the studies (Table 3). There was also little consideration of the cost-effectiveness in the studies in this review. A roll-out of a PI would need to be evaluated, for example with regards to time and cost-effectiveness, particularly where services/resources are limited.

### 4.3 Active components

There were difficulties in analysing and comparing PIs (see ‘limitations’), and deconstructing their components was challenging, particularly when evaluating the elements associated with beneficial effects. As with the review of PIs in adult depression, the mechanism of action was difficult to assess on the basis of the current evidence. Also, for those who are currently depressed and even those at risk, PIs might well be used as an adjunct to established approaches such as CBT, interpersonal therapy (IPT) and/or antidepressant medication. When developing/evaluating PIs, it would be helpful if authors developed a programme theory or logic model which described the mechanism of effect of their intervention, and evaluated this using methods like mediation analyses and/or through the study process evaluation [45].

Personalising the content and taking a person-centred approach might be important in the success of programmes [46]. Beardslee et al. [29] stated that combining a PI with a family’s individual experiences ensured lasting improvements. Parents in some interventions preferred to have the amount/level of information tailored to their needs, so that it was relevant to them. Incorporating personal stories might be particularly helpful [22].

With regards to the specific content of programmes, learning to identify symptoms and plan activities could be important, and information on lifestyle approaches such as exercise. In their RCT of low-level interventions, Parker et al. [20] found that physical activity was most effective in reducing depressive symptoms. In adults, Tursi et al. [11] hypothesised that ‘teaching lifestyle regularity’ may help with the prevention of depression, whereas early detection of prodromal symptoms may be important for preventing relapses.

It is also likely that the success of a programme is related to the way it is delivered. The skills of the facilitator and therapeutic relationship could be key factors [47]. This review showed that facilitated PI could be delivered by a range of professionals, including nurses, psychiatrists, psychologists and health visitors. Colom [9], a pioneer of PIs in mood disorders, particularly in adults, noted that interpersonal skills and ‘common sense’ were especially important facilitator characteristics, and that those delivering PIs need to be an expert on the ‘disorder’, not the ‘technique’. This would avoid the ‘complex training’ and associated funding required, for example for CBT. Furthermore, such skilled approaches are not always available; not only in LMIC, but also in higher income countries, and PIs could help address this need.

The mode of communication, such as the use of multimedia, can also accommodate personal learning styles and preferences [48], and make it more engaging and accessible [21]. Repeating key themes and messages in various ways might also help [29]. Therefore, it might be important that there is a range of formats (modalities, materials, activities) available to deliver PIs.

### 4.4 Strengths, limitations

This review has a number of important strengths; it is the first of its kind exclusively on PIs in adolescent depression, it was conducted rigorously, and efforts were taken to minimise bias, for example through two people completing independent searches and data extraction. However, the findings should be interpreted bearing in mind a number of considerations and limitations.

#### 4.4.1 Heterogeneity, methodological quality

There was a diversity of approaches in the research design and PI approaches in the papers selected, which made it difficult to compare programmes directly and measure the overall effectiveness, and therefore no *meta*-analysis was attempted. The lack of consensus and diverse approaches were difficulties encountered by other reviewers when comparing programmes and studies [11].

The studies targeted a range of participants – young people with depression, those at high-risk, and parents/families. The programmes ranged in format, number and duration of sessions and the use of facilitators. PIs were also not always tested in isolation, and often were incorporated, for example with or versus CBT, and the use of control groups varied. The outcomes related to the individuals or parents/carers, or both, and a range of instruments was applied. Some papers did not describe these elements. All this made comparative analysis difficult.

The studies included were at various stages, from early development/evaluation to efficacy trials. Many had small sample sizes and short-term follow up. It was unclear how many of the programmes were developed using relevant theory, following extensive mixed-methods approaches with user input, and for a wide range of settings or services, in line with recognised frameworks [45]. The review included only published studies, and some studies/programmes might not have been documented or accepted for publication. This review might have been scientifically more robust had it included only RCTs. However, this approach would have yielded few studies, and excluded a number of relevant and interesting programmes, which could help inform work in this field.

The lack of large scale RCTs might be related to the lack of PIs available for adolescent depression in services in general, and the difficulty in setting up large multi-centre experimental designs, because of the time/funding investment required (e.g. to recruit, train practitioners, ensure similar delivery across centres). It might also be related to the ‘branding problem’ of PIs (noted below), and how PIs may be regarded as some as a low-level approach (e.g. compared to ‘skilled’ approaches such as CBT), and perceived as better suited to control groups, rather than the main intervention.

Furthermore, most studies were conducted in high-income countries, and few in less economically developed countries. The more advanced packages were developed in North America, Scandinavia and Australasia − especially e-health interventions. There are differences in each country, for example in service structure, culture and language, and there might be difficulties in implementing them elsewhere. Those in the adolescent age group might be particularly sensitive to such differences. Only English-language studies were selected, and so this may also have introduced bias.

#### 4.4.2 Defining PI

The approach to the definition of psychoeducation was variable in the programmes and studies in the review. There was a lack of clarity on the difference between ‘psychological’, ‘psychoeducational’, and ‘educational’, and the terms were used inter-changeably in some publications [12]. Many programmes reviewed had elements of psychological therapies such as CBT or IPT, and it was difficult at times to separate the psychological and educational components – although there might be some overlap and similarities between the approaches. This was consistent with the description of the ‘blurry’ boundaries between ‘simple’ interventions (e.g. PI) and ‘skilled’ approaches (e.g. CBT) [9].

The distinction between general health information and psychoeducation was also unclear at times, for example in relation to the many printed (leaflets/books) or online resources for young people (e.g. RCPsych, Headspace). Trials have used printed literature with a control group [49]. However, there was little literature on the development and evaluation of such resources. It is possible that the term ‘psychoeducation’ should be reserved for ‘active’ intervention programmes with individualised/tailored information for young people and families, to help prevent and manage difficulties, as opposed to general ‘passive’ resources.

Another limitation of this review was that the searches were for articles with ‘psychoeducation’ (or psychoed*) in the title/abstract. This meant that relevant studies described as ‘educational’ programmes, might have been missed, although the searches went beyond the use of databases. Furthermore, given there are many possible psychosocial risk factors for adolescent depression, PIs that targeted such factors may have been missed.

### 4.5 Conclusions, practice implications

A limited number of PIs were developed and evaluated in line with recognised research frameworks and using rigorous methods of evaluation, and the large variation in approaches made it difficult to evaluate the overall effectiveness of PIs for adolescent depression. However, the findings to date for PIs in adolescent depression show some promise. Although evidence is limited, a range of potential benefits have been reported, from increased understanding and change in behaviour and attitudes, to improved family communication and effects on mood symptoms and wellbeing.

Whilst the evidence for the effectiveness of electronic PI (or e-PI) is limited thus far, many packages are in development [e.g. 35], and online PI in adults has been shown to reduce depressive symptoms and improve understanding of treatments [50]. There is evidence to support the use of CBT-based and other online packages [51], [52]. The use of social media, technology (e.g. smartphones), and online material has been identified as a key area of future practice/research in adolescent depression [8]. However, there are also challenges, related to data protection, privacy and security, and the ‘digital divide’ between those who have access to the internet and those who do not, although this gap is narrowing [53].

Future work should include defining ‘psychoeducation’ in international guidelines, to help remedy the ‘branding problem’ referred to by Colom [9]. This would not only help with future research (including reviews) in the field, but also help to clarify to individuals, families/carers and professionals what PIs might entail, how PIs might help them, and how they compare with other approaches. However, the definition should not be too restrictive, and acknowledge there may be some overlap and similarities with other approaches/therapies, and embrace the range of possible formats of PIs.

Programmes should also be developed/evaluated according to recognised research frameworks [45]. Further research is required testing PIs in adequately powered (possibly multi-centre) RCTs, possibly alongside other therapies such as CBT, with process and economic evaluations included as part of the trial. There needs to be more emphasis on the theory, content and design of interventions, and an exploration of the active components of PIs, and potential mechanisms of PIs through process evaluation and investigations such as mediation analyses.

Further research is also needed to understand how to personalise the information and design, and incorporate multi-modal approaches, given the variety of experiences of depression. Programmes need to accommodate and engage with a range of ages, backgrounds and abilities – particularly when motivation and concentration is impaired during depressive episodes. Future studies could explore how PIs could be integrated into the daily lives of young people and families/carers, and into health, social, education and youth services.
